# A remarkable new genus and species of Nemourinae (Plecoptera, Nemouridae) from Sichuan, China, with systematic notes on the related genera

**DOI:** 10.1371/journal.pone.0229120

**Published:** 2020-03-04

**Authors:** RaoRao Mo, Maribet Gamboa, Kozo Watanabe, GuoQuan Wang, WeiHai Li, Ding Yang, Dávid Murányi

**Affiliations:** 1 Guangxi Key Laboratory of Agric-Environment and Agric-Products Safety, Agricultural College, Guangxi University, Nanning, Guangxi, China; 2 Department of Plant Protection, Henan Institute of Science and Technology, Xinxiang, Henan, China; 3 Department of Civil and Environmental Engineering, Ehime University, Matsuyama, Japan; 4 Department of Entomology, China Agricultural University, Beijing, China; 5 Department of Zoology, Eszterházy Károly University, Eger, Hungary; 6 Department of Zoology, Hungarian Natural History Museum, Budapest, Hungary; Laboratoire de Biologie du Développement de Villefranche-sur-Mer, FRANCE

## Abstract

A remarkable new genus and species of Nemourinae, *Sinonemura balangshana* gen. et sp. n., is described from Balang Mountains, Sichuan, southwestern China. The description is based on morphology and molecular data. The Nemourinae genera related to the new taxon are re-evaluated on the basis of comparative functional morphology of male epiproct. Notes on the Asian distribution of the Nemourinae are also given.

## Introduction

The forestfly genus *Nemoura* Latreille, 1796 [1] is one of the most species-rich genera among the stoneflies (Plecoptera), and is widely distributed in running waters of Holarctic and Oriental regions. Approximately 200 valid species are known [2]. During the 19th century, almost all Nemouridae species were included in this genus, along with many other species of different stonefly groups (even species that belong to Antarctoperlaria a second suborder of the Plecoptera). At the turn of the 20^th^ century, Peter Kempny’s new diagnosis of the genus and the descriptions of two subgenera [3], brought the supraspecific classification of the Nemouridae into a debate. During the following decades, the genus *Paranemoura* Needham & Claassen, 1925 [4] and ten further subgenera [5,6] were described, and Illies [7] elevated all twelve subgenera of *Nemoura* to generic level. However, *Nemoura* remained as a poorly defined assemblage with paraphyletic taxa in agreement with molecular analyses [8].

A generic revision of the Nemouridae was performed in the seventies [9] and the family was divided in two well supported subfamilies. Among these, the proposed generic system of the Amphinemurinae became accepted and later completed with the descriptions of two additional genera [10,11]. In the Nemourinae, the less speciose and well defined group of genera that lack lateral knobs on the male epiproct raise few questions of higher classification. However, delimitation of the Nemourinae genera that display lateral knobs on the male epiproct still pose several unanswered questions in stonefly systematics. At present, three genera with lateral knobs are distinguished. Among them, the validity of *Illiesonemoura* Baumann, 1975 [9] has been challenged [12]. *Zapada* Ricker, 1952 [6] was considered a Nearctic genus whose presence in the Palaearctic was just recently recognized [13]. And *Nemoura* contains many species or species groups that differ from the concept of the genus (e.g. [14–16]).

Currently, the subfamily Nemourinae includes 13 known genera, 264 valid species, and five extinct species [2]. A total of 42 species of two genera, *Illiesonemoura* and *Nemoura*, are known from China and have been previously studied by Chen & Du [17,18], Chu [19], Du, Zhou & Wang [20], Illies [7], Kawai [21], Klapálek [22,23], Li & Yang [24–29], Li, Feng & Yang [30], Li, Wang *et* Yang [31], Li, Murányi, Pan & Yang [32], Qian, Qian, Chen & Du [16], Shimizu [33], Sivec [34], Sivec & Stark [35], Uéno [36], Wang & Du [37], Wang, Du, Sivec & Li [38], Wang, Li & Yang [39], Wu [40–47], Yang, Li & Zhu [48], Zhiltzova [49], Zhu & Yang [50], Zwick [51–53].

In August 2013, a male and female of a peculiar new nemourid were collected in Sichuan Province of Southwest China. We determined that an assignment of this new species to either *Nemoura*, *Illiesonemoura*, or *Zapada* would necessitate broadening of morphological diagnoses of these genera. Morphological studies are based on previously published extensive molecular analysis [8]. In addition to raise several other questions about the systematics of the Nemourinae, the molecular studies already supported the new species as a well separated lineage within the related genera [8]. Herein, we describe a new genus, and compare to other Nemourinae that possess lateral knobs and a basal cushion on the epiproct.

## Materials and methods

### Ethics statement

All type specimens of the new taxa described herein, were collected in a governmental, unprotected area (Balangshan, Sichuan Province, China), where no permission was required. Specimens used as comparative materials (S1 File) were acquired from authorized museum collections. Species of the family Nemouridae are not protected in the countries where the type materials or the comparative materials originated.

### Material examined

The type specimens were collected by aerial net and preserved in 75% ethanol. All specimens are deposited at the Entomological Museum of China Agricultural University, Beijing (CAU), the Henan Institute of Science and Technology, Xinxiang (HIST) and the Hungarian Natural History Museum, Budapest (HNHM). Collecting event are provided for all these specimens in the S1 File. Specimens were examined with the aid of a Leica M420 dissecting microscope. Drawings were made with the aid of a drawing tube, and the color illustrations were made with the aid of Imaging Source CCD attached to Leica S8APO. Both male and female terminalia were cleared in 10% KOH solution for ten minutes and dissected with insect pins for the study of internal structures, e.g. sperm duct or oviduct. Illustrations on the male epiproct structures (Figs 3, 5 and 6) were compiled from draft drawings from the intact KOH cleared specimens and from dissected genitalia.

Terminology follows Baumann [9], Zwick [53] and Zwick & Baumann [54] with modifications. The morphological analysis was performed on the dissected genitalia of KOH cleared specimens. Molecular studies and analysis related to the present work were published by Gamboa et al. [8] and the sequences were deposited to GenBank (accession number: MK132446, MK132447).

Maps presented in Fig 7 are original drafts prepared on semitransparent paper and figures were finished by Adobe Photoshop CC 2019. Administrative borders and elevation lines above 2000 meters were drew on the semitransparent paper based on maps on pages 102–103 and 117 in The World Atlas, Second Edition, Chief Administration of Geodesy and Cartograhy Under the Council of Ministers of the USSR, Moscow, 1967. Current administrative division of Sichuan Province was corrected manually.

### Nomenclatural acts

The electronic edition of this article conforms to the requirements of the amended International Code of Zoological Nomenclature, and hence the new names contained herein are available under the Code from electronic edition of this article. This published work and their nomenclatural acts have been registered in ZooBank, under the ZooBank LSIDs (Life Science Identifiers, http://zoobank.org/; LSID: urn:lsid:zoobank.org:pub: 2F1F7055-DB5D-4F93-AEB9-0CF26287F717).

## Results

### Taxonomy

#### *Sinonemura* Mo, Li & Murányi gen. n

urn:lsid:zoobank.org:act: F01C58CA-DA54-4920-AF39-5DB3E651143D.

**Type species.** *Sinonemura balangshana*, new species (here designated by monotypy).

**Diagnosis.** Both sexes have a unique pair of unbranched, finger-like gills on both sides of the cervical sclerite. Male tergum X consists of a divided medio-apical plate armed with a mediobasal projection. Male epiproct with basal cushion, lateral knobs and rows of ventral spines, lateral arms run from apex over midlength, ring of ventral sclerite lacks apical sclerites but connected to parallel medial sclerites. Female is defined by the presence of large and bilobed pregenital plate, raising from posterior margin of sternum VII and reaching sternum IX. Subgenital plate needle-like, with large vaginal lobes; inner sclerite lacking.

**Description.** Small-sized Nemourinae. Cervical gills present, consisting of single gill on each side of the cervical sclerite. Macropterous in both sexes; sexual dimorphism only in size and genitalia. Setation generally short and dense, longer on terminal segments. General color dark besides pale basal abdominal segments, forewings distinctly mottled. Legs of usual length for the family, tibiae longer than femora; claws symmetrical, arolium small.

**Fig 1:**
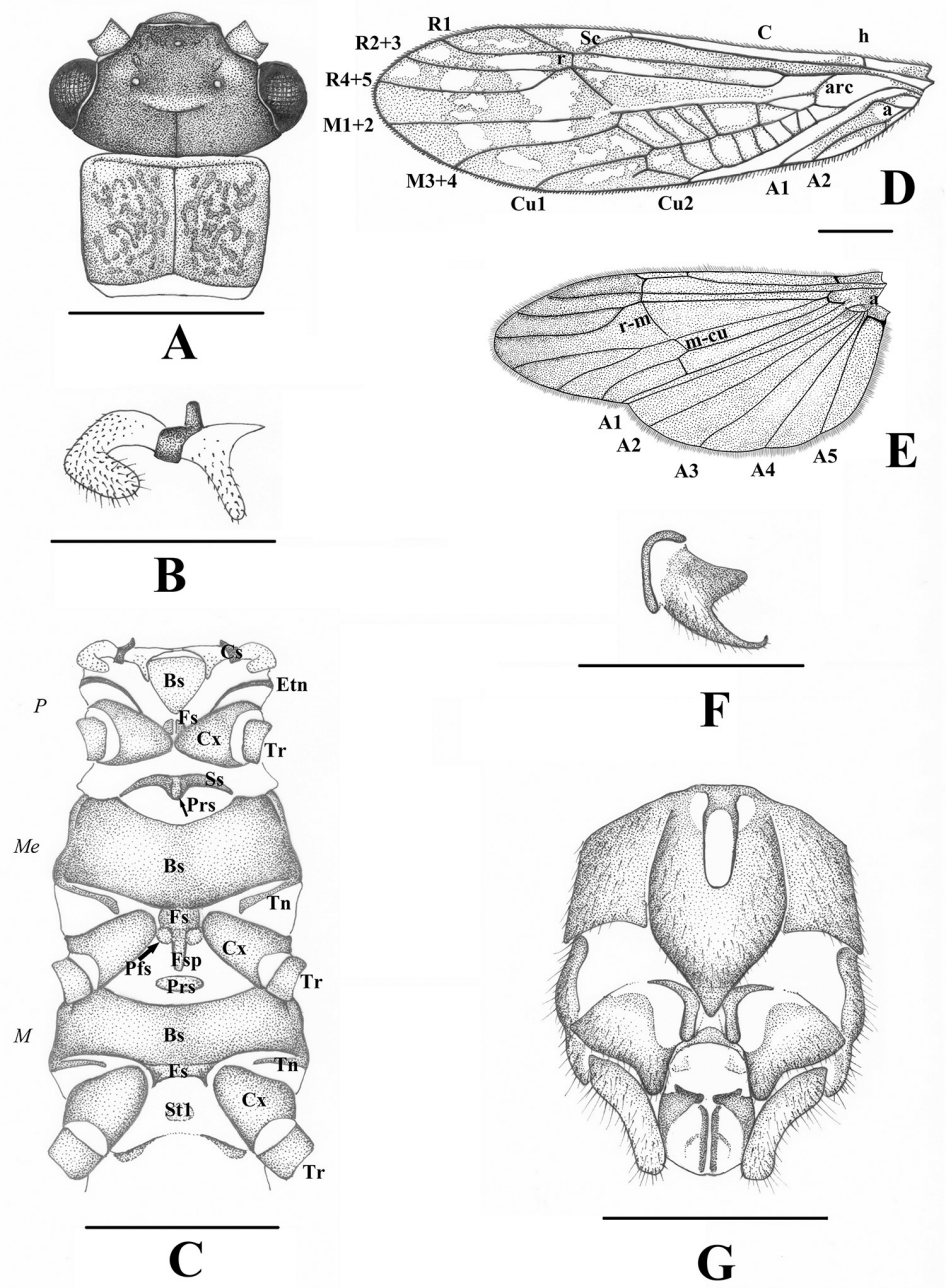
*Sinonemura balangshana* gen. n., sp. n. (A) Head and pronotum, dorsal view. (B) Gills, ventral view. (C) Ventral sclerites of thorax. (D) Forewing. (E) Hindwing. (F) Male paraproct, lateral view. (G) Male terminalia, ventral view. Scale 1 mm for Figs A, C-E, 0.5 mm for Figs B, F-G. Abbreviations: Figs D-E: A: anal vein; a: anal crossvein; arc: arculus; C: costa; Cu: cubital vein; h: humeral crossvein; M: medial vein; m-cu: mediocubital crossvein; R: radial vein; r: radial crossvein; r-m: radiomedial crossvein; Sc: subcosta; Fig C: *P*: prothoracic; *Me*: mesothoracic; *M*: metathoracic; Bs: basisternum; Cs: cervical sclerite; Cx: coxa; Etn: eutrochantin; Fs: furcasternum; Fsp: furcasternal pit; Pfs: postfurcasternum; Prs: presternum; Ss: spinasternum; St1: Sternite 1; Tn: trochantin; Tr: trochanter.

Ventral sclerites of thorax (Fig 1C): prothorax with backwards directed single gill on either side of cervical sclerites; outer gill curved and robust while inner gill thin and straight, both with setae (Fig 1B). Basisternum triangular, lightly sclerotized and separated from other sclerites; eutrochantin stripe-like, furcasternum small and divided, partially covered by the large coxae, postfurcasternum lacking. Mesothorax with stripe-like spinasternum fused with presternum, not touching the large basisternum; trochantin stripe-like. Furcasternum rectangular, fused with basisternum and continuing in large furcasternal pit; paired postfurcasternum small and rounded, fused with both furcasternum and furcasternal pit. Metathorax with small and lightly sclerotized presternum, spinasternum is lacking; basisternum large, trochantin stripe-like. Furcasternum transverse, fused with basisternum; furcal pit and postfurcasternum are lacking.

Wing venation (Fig 1D and 1E): forewing at cord clearly with an X-shaped pattern typical of the family. Forewing C strong and setose; Sc run close to C at apical third, joins R before r. Single crossvein between C and Sc besides h; h equidistant distance between wing base and arc. R1 slightly curved, terminal costal crossvein is joining R after r. R2+3 and R4+5 branching shortly after r. M branches out to M1+2 and M3+4 with r-m nearly joining to r; r-m and M1+2 are interrupted near junction. Five or six crossveins between M and Cu1 in addition to arc and m-cu. Crossveins between Cu1 and Cu2 numerous; Cu2 ends before 1/2 of wing length, cu-a lacking. A1 ends near branching of R, slightly curved beyond a; A2 reaches as far as arc. Hindwing C, Sc, R and crossveins similar to the forewing but the X-shaped pattern lacking. M branching to M1+2 and M3+4 far after r-m; M1+2 is more backwards directed than on forewing; No crossvein between M and Cu1 between arc and m-cu. Single crossvein between Cu1 and Cu2 after arc; Cu2 parallel to A1 and ending beyond r, cu-a lacking. Anal field large, the fold of the wing extending between the parallel Cu2 and A1. All A veins straight; A1 reaches about the same distance as Cu2, A2 ends in r, A3 reaches near Sc, A4 ends halfway between h and r, and A5 reaches near arc. Longitudinally directed a reaches base of A5 that gives a basal crossvein ventrally.

**Fig 2:**
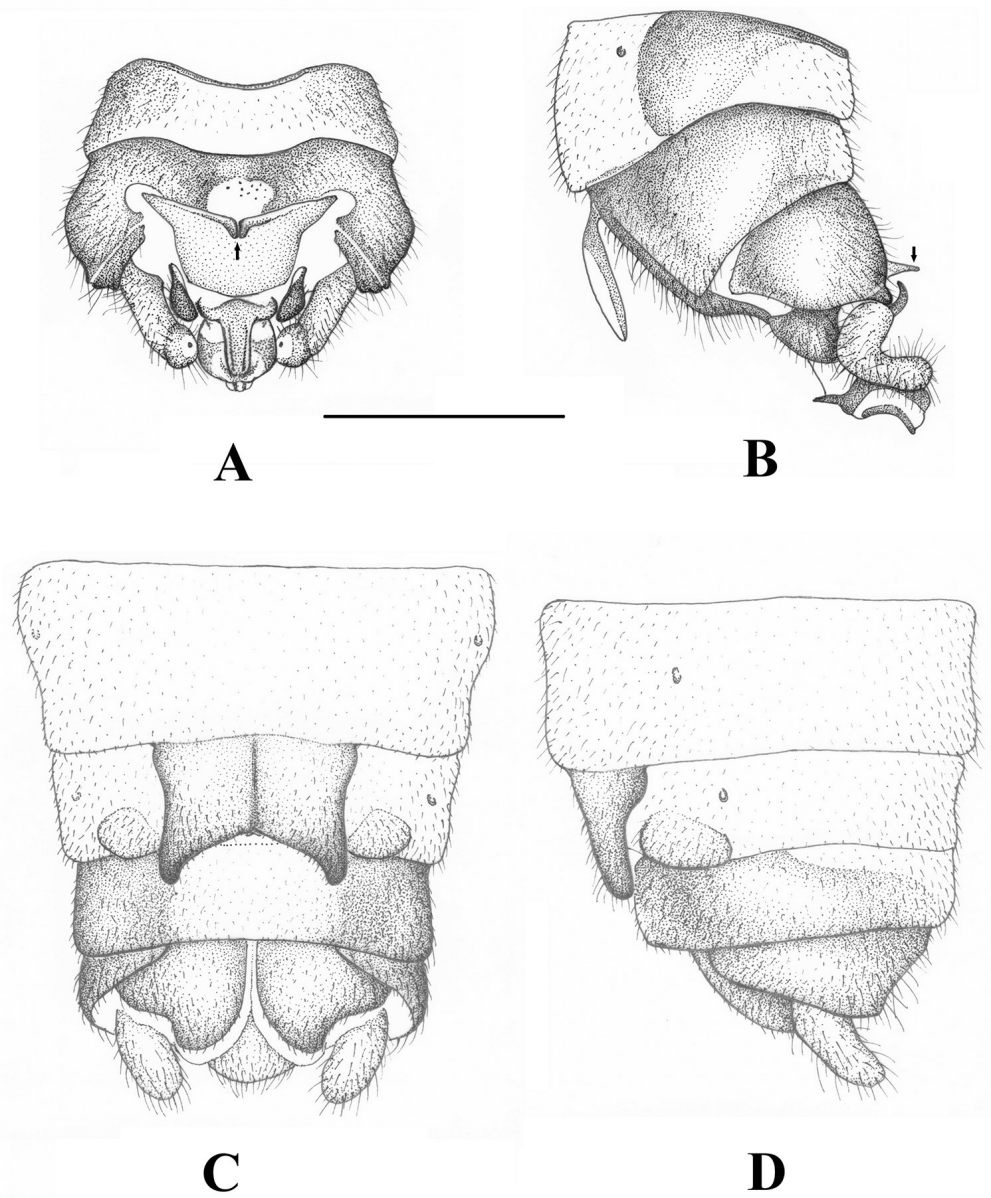
*Sinonemura balangshana* gen. n., sp. n. (A) Male terminalia, dorsal view. (B) Male terminalia, lateral view. (C) Female terminalia, ventral view. (D) Female terminalia, lateral view. Scale 0.5 mm. Arrow indicates mediobasal projection of medioapical plate of Tergum X.

**Fig 3:**
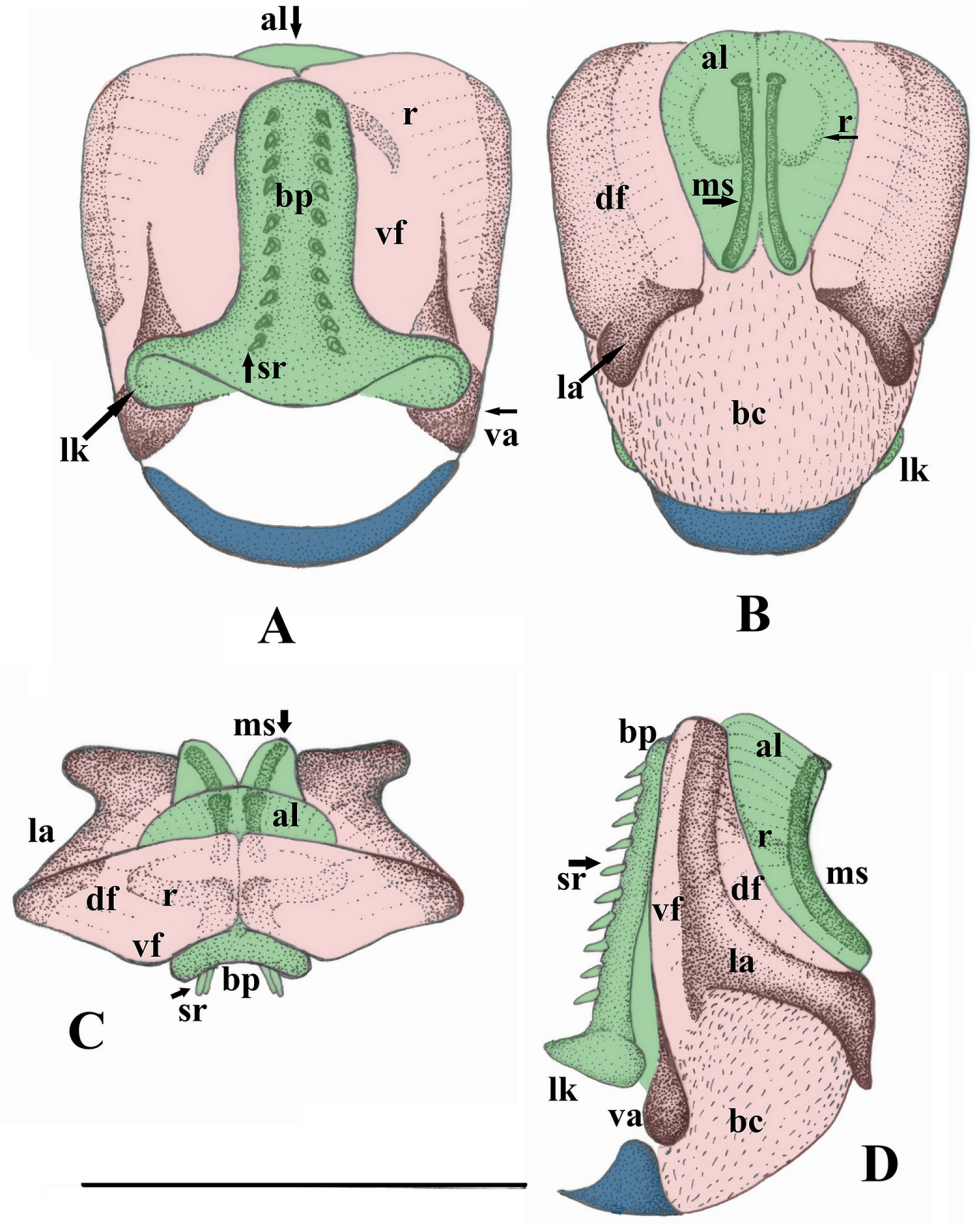
*Sinonemura balangshana* gen. n., sp. n. (male epiproct) (A) Ventral view. (B) Dorsal view. (C) Caudal view. (D) Lateral view. Scale 0.5 mm. Abbreviations: al: apical lobe; bc: basal cushion; bp: basal plate; df: dorsal fold; la: lateral arm; lk: lateral knob; ms: medial sclerite; r: ring; sr: spine row; va: ventral arm; vf: ventral fold. Colors: blue: basal sclerite; green: ventral sclerite; pink: dorsal sclerite.

Male abdomen (Figs 1F, 1G, 2A, 2B and 3): terga I–IX without notable modification, but dark lateral sclerites and entire antecosta present and increasing in size towards the apical segments. Tergum X with small medial membranous field, medio-apical portion separated to a distinct bald plate that bears mediobasal projection; the projection weakly separated medially. Sterna I–VIII without notable modification. Hypoproct typical of Nemouridae, bearing an elongated vesicle. Paraproct inner lobe simple, well separated from large outer lobe that ends in an acute, hook-like apex. Cercus elongated and lightly sclerotized, lacking hooks or other modifications. Epiproct with large basal cushion above small basal sclerite, lateral arms run from the apex over midlength and end in strongly sclerotized horns. Ventral sclerite with small lateral knobs and two parallel rows of ventral spines. Ring of the ventral sclerite lacks apical sclerites but connected to two parallel sclerites, apically elevated above the dorsal sclerite.

Female abdomen (Fig 2C and 2D): terga I–IX unmodified, tergum X entirely sclerotized; epiproct separated from tergite X, simple and rounded. Sterna I–VI without notable modification. Sternum VII with large, bilobed pregenital plate arising from the posterior margin of the segment, overhanging entire sternum VIII and reaching anterior portion of sternum IX. Sternum VIII with small, needle-like subgenital plate totally covered by pregenital plate, and bear two large, completely hairy vaginal lobes. Sternum IX fully sclerotized but with lightly colored medial field. Sternum X unmodified, paraproct with blunt tip; cercus stout, unmodified.

**Fig 4:**
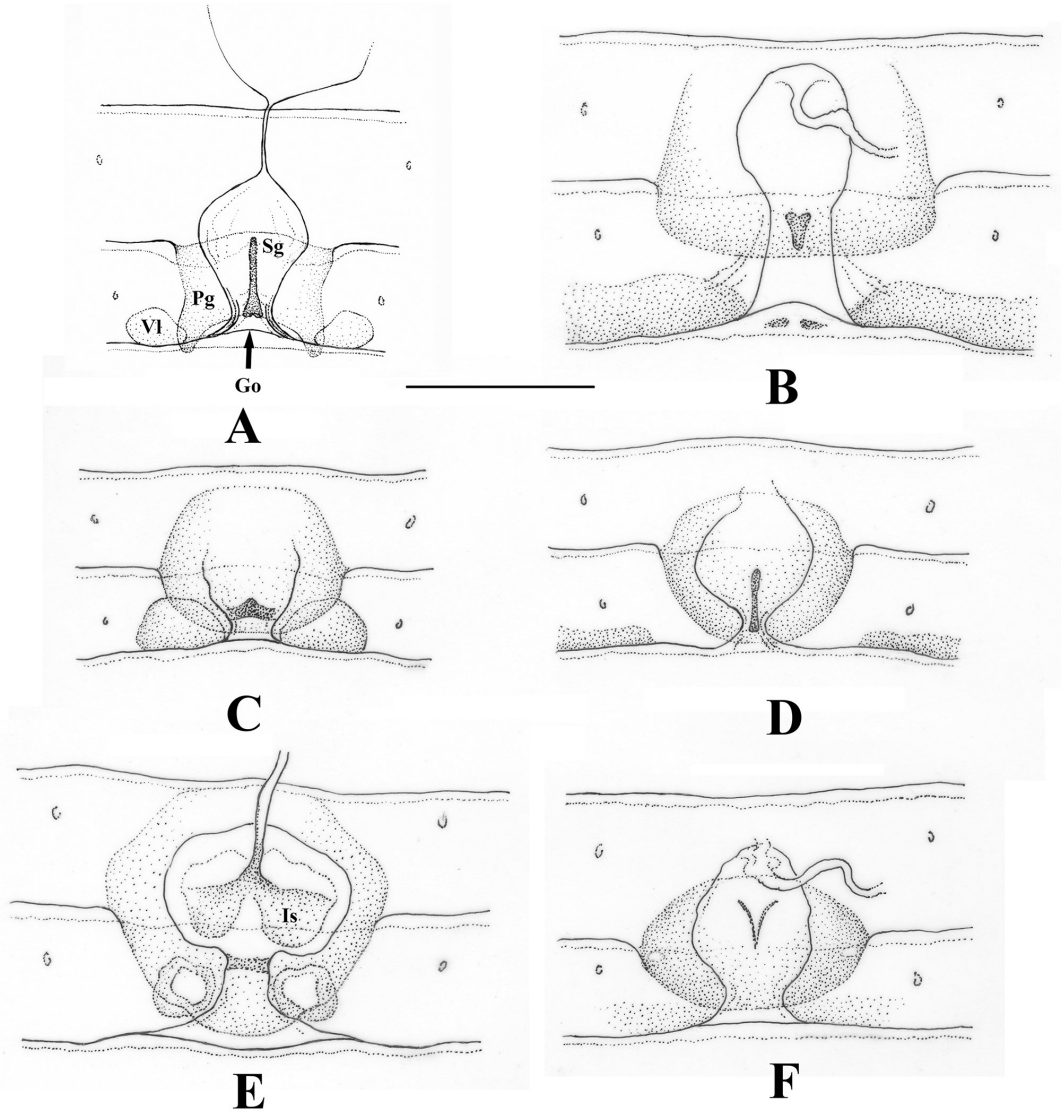
Female inner genitalia of Nemourinae genera. (A) *Sinonemura balangshana* gen. n., sp. n. (B) *Zapada cinctipes* (Banks, 1897). (C) *Illiesonemoura pakistani* (Aubert, 1959). (D) *Illiesonemoura polystigma* (Aubert, 1959). (E) *Nemoura cinerea cinerea* (Retzius, 1783). (F) *Nemoura flexuosa* Aubert, 1949. Scale 0.5 mm. Abbreviations: Go: Genital opening; Is: Inner sclerite; Pg: pregenital plate; Sg: subgenital plate; Vl: vaginal lobe.

Female inner genitalia (Fig 4A): genital opening wide, positioned at posterior margin of the pregenital plate. After short narrowing section, genital opening continued in wide, membranous atrium but lacks inner sclerite. The atrium continuing in a short ductus connected to a large, membranous spermatheca.

Larva: unknown.

**Comparative notes.** The structure of the gills, the wings, the male and female genital characters place the new genus within the subfamily Nemourinae, in the sense of Baumann [9]. The molecular analysis indicated the new genus belonged to the paraphyletic assemblage of *Nemoura* sensu lato. The specimens of *Illiesonemoura* were not available for molecular studies [8]. Thus, the phylogenetic position is not completely clear, because similarities of male genitalia would place the new genus close to the *pakistani* group of *Illiesonemoura*.

The presence of one unbranched gill on both sides of cervical sclerites is a character typical for adults and larva of the Nemourinae genus *Zapada*, and also for the Amphinemurinae genus *Sphaeronemoura* Shimizu & Sivec, 2001 [10]. However, gills of these genera are of the same size on both sides of the cervical sclerites, while outer gill of the new genus is curved and more robust than the thinner, straight inner gill (Fig 1B).

The wing venation of the new genus is typical for the family and shows no any special characteristics. A distinctly mottled wing patterns was observed in the new genus (Fig 1D). These patterns are only common within the genus *Illiesonemoura*, and characteristic for some Asian *Nemoura*, *Amphinemura* (Ris, 1902) [5] and *Protonemura* Kempny, 1898 [3] species groups among the Palaearctic Nemouridae. The Nearctic genera *Prostoia* (Ricker, 1952) [6] and *Shipsa* (Ricker, 1952) [6], Nearctic species of *Zapada* and some southwestern Nearctic species of *Amphinemura* also shown mottled or striped wing patterns. The Asian Nemouridae with mottled wings are all high montane insects, with the exception of the *A*. *megaloba* species group from Japan [55].

The female terminalia superficially resembles to the Amphinemurinae, as shape of the apically positioned pregenital plate is similar to the subgenital plate of most Amphinemurinae genera (Fig 2C). However, it is just an unusual modification and the pregenital plate entirely covers a well-developed subgenital plate that can be seen only in cleared specimens from the ventral surface (Fig 4A). The shape of the subgenital plate is similar to the Holoarctic genus *Podmosta* [6, 56]. Presence of large vaginal lobes, together with exceptionally enlarged and apically positioned pregenital plate and the long but narrow subgenital plate readily distinguish the female from all other known Nemouridae, with no similar female genitalia structure apparent in other known genera.

The presence of lateral knobs and row of spines on the ventral sclerite of male epiproct would place the new genus close to the genera *Nemoura*, *Illiesonemoura*, and *Zapada*. Among these genera, the male terminalia is most similar to those of the *I*. *pakistani* species group sensu Aubert [57], as the presence of two parallel stick-like sclerites attached to the apex of the ring of ventral sclerite, lacking an apical sclerite, and the paraproct’s outer lobe terminates in a hook-like apex (Fig 3). This group was described in *Nemoura* sensu stricto, later transferred to *Illiesonemoura*, but Zwick & Sivec [12] questioned the validity of that genus and described further species of the group assigned to *Nemoura*. The new genus can be distinguished from the *pakistani* species group by the apically extending lateral arm of the epiproct and completely separated lobes of the paraproct (Figs 1F and 3D). In addition, complete separation of medio-apical portion of tergum X that bears distinct mediobasal projection can be found only in case of a single member of the *pakistani* species group, *N*. *unicornis* Jewett, 1975 [58], but that species lacks the single gills, characteristic of other species in the *pakistani* species group, and having shortened, uniformly brown wings. Besides the *pakistani* species group and the typical species assigned to *Illiesonemoura*, the male terminalia of the new genus differs from sensu stricto *Nemoura* males (based on the type species, *N*. *cinerea* (Retzius, 1783) [59], and most European, Asian and North American species) by lacking apical sclerites of the epiproct and lacking cercal hooks. As for *Zapada*, it can be distinguished by the short ring of the ventral sclerite of the epiproct and by the elongated cercus.

**Etymology.** The genus name is derived from the composition of the words Sino and *Nemoura*. The first refers to China, as the hitherto known single species is probably endemic to the country, while the second correspond to the family where the new genus belongs. *Nemoura* is simplified as *nemura*, to prevent homonymy with the doubtful fossil genus *Sinonemoura* Ping, 1928 [60]. Used as a noun, gender feminine.

**Remarks.** The female and larva of a probable congener were found in Sichuan Province by the colleague Heather Fair, Ohio State University (pers. comm.). Ongoing molecular and morphological studies will clarify if the specimens belong to *Sinonemura*.

#### *Sinonemura balangshana* Mo, Li & Murányi sp. n

urn:lsid:zoobank.org:act: 767E761A-2668-4420-B028-49D864245D21.

**Type material** (1 ♂, 3 ♀). Holotype ♂ (CAU), CHINA, Sichuan Province, Tibetan Qiang Autonomous Prefecture of Ngawa, Wenchuan County, Balangshan, N 30.89905, E 102.91166, 3993m, 2013.VIII.6, Leg. X.Y. Liu. Paratypes: 2 ♀ (HIST), 1 ♀ (HNHM), same data as holotype.

**Diagnosis.** As for the genus.

**Description.** Macropterous in both sexes, measurements: male holotype forewing length 6.2 mm; female paratypes forewing length 7.8 mm. Head uniformly black, tentorial callosities and M-line hardly visible; scape and pedicel dark brown, rest of antenna and palpi brown (Fig 1A). Pronotum brown with numerous dark brown rugosities; wider than long, shape rectangular but with edges rounded (Fig 1A). Other sclerites of the thorax are evenly dark brown. Femora brown, apical portion darker; tibiae brown in their basal 1/5, rest are light brown. Tarsi and claws are brown. Forewing with distinct mottled pattern, hindwing hyaline besides upper portion of its apex that is darker; venation dark brown (Fig 1D and 1E).

Male abdomen: terga I–II unmodified, terga III–VIII with paired anterolateral dark sclerites increasing in size towards apical segments; antecosta entire on terga VI–VIII. Tergum IX similar to previous terga but lateral sclerites joined to the well sclerotized sternum IX (Fig 2A). Tergum X with entire antecosta, medial fifth of the segment with a rounded membranous field that bear short sensilla-like setae; the field asymmetrical on the only available male (Fig 2A). Medio-apical portion of tergum X is separated to a large, bald plate that bear paired mediobasal projection (Fig 2A); the projection appears as a single large spine in lateral view (Fig 2B). Sternum I with small medial sclerite, sterna II–VIII with small, paired anterolateral sclerites. Hypoproct one and 1/2X longer than wide, oval, apical part gradually tapering and blunt; vesicle elongated, 3X longer than wide (Fig 1G). Paraproct inner lobe L-shaped, dark brown and well separated from outer lobe (Fig 1F and 1G). Outer lobe large and with wide base, inner portion of the base lightly sclerotized; apical half dorsally curved around the cercal base, ends in an acute apex that bears small, membranous inner lobe (Figs 1F and 2A). Cercus light brown, elongated and strongly dorsal curved; apex slightly bulbose, terminal wart can be seen only from dorsal view (Figs 1G, 2A and 2B). Epiproct short and broad in dorsal, high in lateral view (Fig 3). Dorsal sclerite with large basal cushion above the small basal sclerite, pair of dark, apically elongated ventral arms delimitate it ventrally; surface of the basal cushion is covered with sharp triangular scales (Fig 3A, 3B and 3D). Lateral arms originate from the apex of dorsal sclerite and run lateral sides of the epiproct over midlength (separating the ventral and dorsal folds), then directed dorsal and end in strongly sclerotized horns, pointed outwards and backwards (Fig 3B, 3C and 3D). Ventral sclerite basally with pair of small lateral knobs, after the broad base continued in a stout basal plate that bears two parallel rows of ten stout spines (Fig 3A). Apically continued in short and thin ring; the ring lacks apical sclerites but dorsal tip of the two arms of the ring connected to a pair of parallel medial sclerites elevated above the dorsal lateral arms of the sclerite and dorsal fold in the apical third of the epiproct, and stretching the apical lobe (Fig 3B, 3C and 3D). These medial sclerites are stick-like, curved in lateral view and their basal end are dorsal raised.

Female abdomen: terga I–IX unmodified, tergum X entirely sclerotized, dark brown; epiproct separated from tergite X, simple and rounded. Sternum I with small medial sclerite, sterna II–III mostly membranous but with paired anterior sclerites, sterna IV–VI unmodified. Sternum VII with large, bilobed pregenital plate that is raising from the posterior margin of the segment, besides pregenital plate the sternum is membranous (Fig 2C and 2D). The pregenital plate is overhanging all sternum VIII and reaching anterior portion of sternum IX; width of the plate is half of segment VIII’s width. Lateral sides of pregenital plate sinuous, lobes are slightly diverging, separated by a wide V-shaped indention; lobes and sides of the plate are dark brown, medial portion light brown. Sternum VIII with small, needle-like subgenital plate totally covered by pregenital plate, seen as a longitudinal dark stripe by transparency; two large, light brown, completely hairy and spherical vaginal lobes set by the sides of the overhanging pregenital plate (Fig 2C and 2D). Sternum IX fully sclerotized but with lightly colored medial field, anteriorly delimited by the overhanging lobes of the pregenital plate (Fig 2C). Sternum X unmodified, dark brown; paraproct wide and with blunt tip, cercus stout, lightly sclerotized.

Female inner genitalia (Fig 4A): inner tip of vaginal lobes is positioned beneath the overhanging pregenital plate. Genital opening wide and intruding far from the lobes inner tip, ventrally connected by the slightly forking posterior margin of the needle-like subgenital plate. After short and laterally well delimited narrowing section, genital opening continued in a wide, membranous atrium that is situated beneath anterior half of segment VIII and posterior half of segment VII. The atrium is continuing in a short ductus connected to a large, membranous spermatheca, situated above sterna V–VI.

Larva: unknown.

**Comparative notes.** As for the genus.

**Etymology.** The new species is named after its type locality, Balangshan, Sichuan Province, China. Used as a possessive noun, gender feminine.

**Distribution and ecology.** China (Sichuan Province). Known only from the type locality, a high mountain area in the Balangshan Range. The specimens were collected on tall herbs and bush, probably emerged from small brooks nearby.

**Key to the adults of the genera of the *Nemoura* genus group.** The following keys are for males and females of *Nemoura* genus group as defined above. The genus *Nemoura* includes only the *Nemoura* sensu stricto group based on the type species, *N*. *cinerea*.

**Fig 5:**
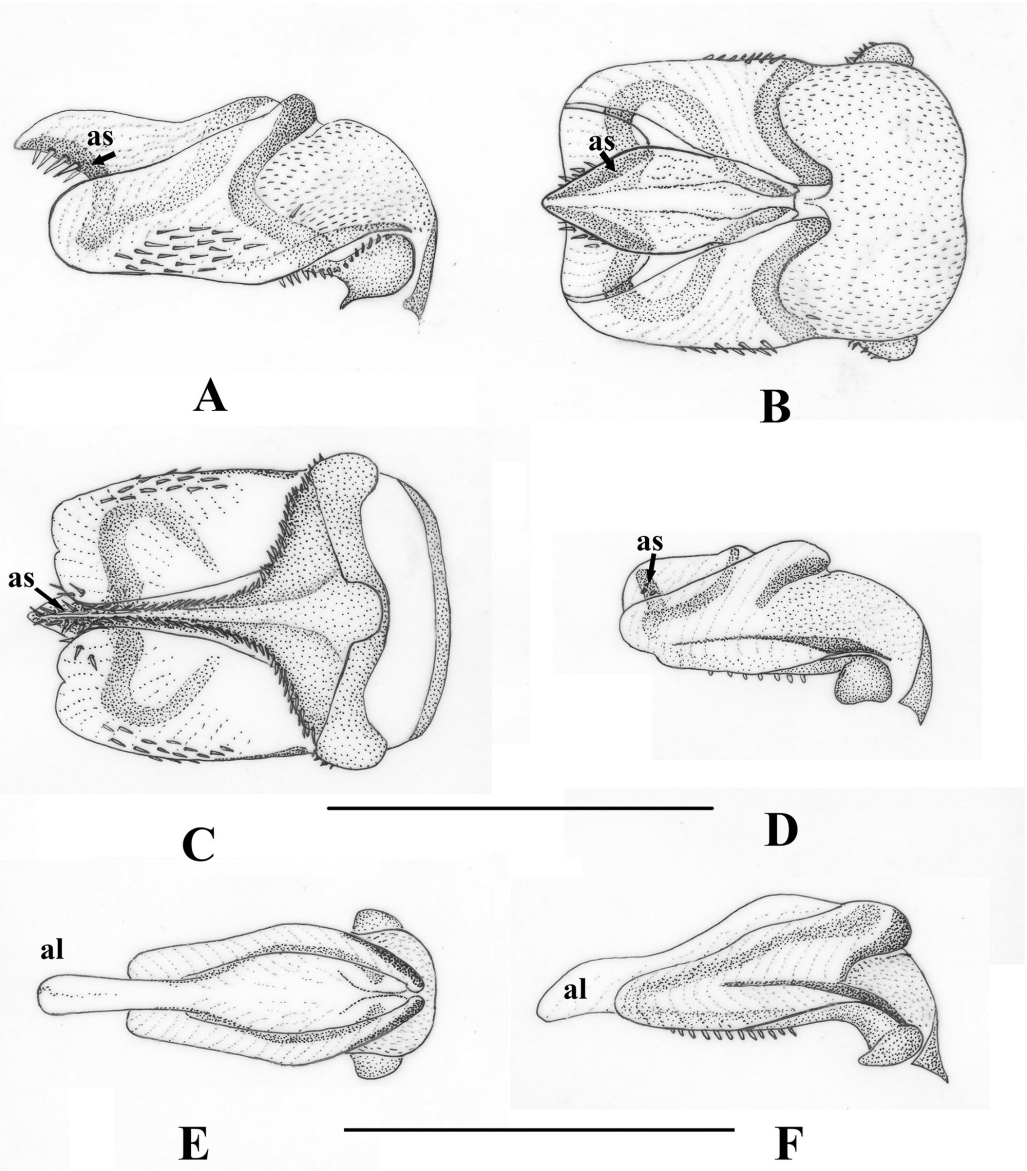
Male epiproct of genera *Nemoura* Latreille, 1796 s.s. and *Zapada* Ricker, 1952. (A) *Nemoura cinerea cinerea* (Retzius, 1783), lateral view. (B) same, dorsal view. (C) same, ventral view. (D) *Nemoura flexuosa* Aubert, 1949, lateral view. (E) *Zapada cinctipes* (Banks, 1897), dorsal view. (F) same, lateral view. Scale 0.5 mm for Figs A-D, 0.25 mm for Figs E-F. Abbreviations: al: apical lobe; as: apical sclerite.

**Fig 6:**
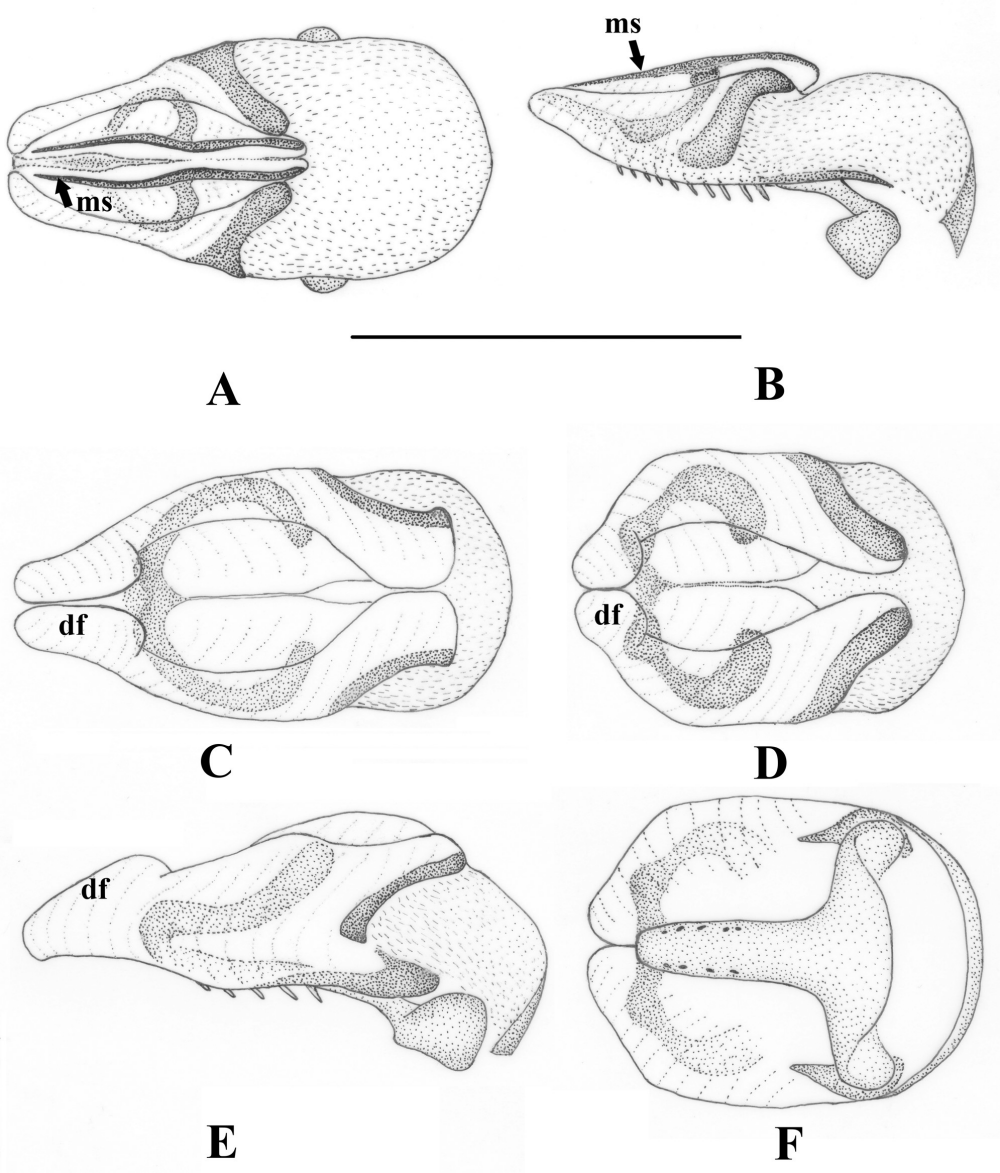
Male epiproct of genus *Illiesonemoura* Baumann, 1975. (A) *Illiesonemoura lilami* (Aubert, 1959), dorsal view. (B) same, lateral view. (C) *Illiesonemoura battakundi* (Aubert, 1959), dorsal view. (D) *Illiesonemoura polystigma* (Aubert, 1959), dorsal view. (E) *I*. *battakundi*, lateral view. (F) *I*. *polystigma*, ventral view. Scale 0.25 mm. Abbreviations: df: dorsal fold; ms: medial sclerite.

**Males**
1. Paraproct divided into inner, median and outer lobes. Lobes can be armed with spines or prongs.        Amphinemurinae (not keyed further)
  - Paraproct undivided, or divided into inner and outer lobes only. Lobes can have apical hooks but never armed with spines.        Nemourinae 2
2. Epiproct lacking lateral knobs and a basal cushion. If vestigial knobs and cushion present, then the epiproct asymmetrical.       .*Lednia*, *Nanonemoura*, *Nemurella*, *Ostrocerca*, *Paranemoura*, *Podmosta*, *Prostoia*, *Shipsa*, *Soyedina*, *Visoka*
  - Epiproct symmetrical, with lateral knobs and basal cushion. *Nemoura* genus group.        3
3. Epiproct with apical sclerite (Fig 5A–5C). Cercus usually with apical hook.        *Nemoura* s.s.
  - Epiproct lacking apical sclerite. Cercus lacking apical hook.       .4
4. Cercus shorter or just slightly longer than wide. Ring of the ventral sclerite elongated (Fig 5E and 5F).       .*Zapada*
  - Cercus at least twice longer than wide. Ring of the ventral sclerite rounded.        5
5. Epiproct having medial sclerite, and the lateral arm is apically extending (Fig 3). Paraproct lobes completely separated.        *Sinonemura*
  - Epiproct lacks medial sclerite, or the lateral arm is not extending apically (Fig 6). Paraproct lobes basally fused.       .*Illiesonemoura*

**Females**
1. Subgenital plate usually distinct and large, if moderately developed, then cervical gills highly branched.        Amphinemurinae (not keyed further)
  - Subgenital plate usually small, if well developed, then gills reduced to small nubs.        Nemourinae 2
2. Pregenital plate usually poorly developed, if well developed, then with distinct vaginal lobes, submental gills or forewing veins A1 and A2 joined.        *Lednia*, *Nanonemoura*, *Nemurella*, *Ostrocerca*, *Paranemoura*, *Podmosta*, *Prostoia*, *Shipsa*, *Soyedina*, *Visoka*
  - Pregenital plate distinct and large. If vaginal lobes present, then partly covered by subgenital plate. Lacks submental gills, forewing veins A1 and A2 not joining.        *Nemoura* genus group 3
3. Small subgenital plate present (usually covered by pregenital plate but seen in cleared specimens).        4
  - Lacks subgenital plate. Vaginal sclerites usually present, vaginal lobes may present (Fig 4E).        *Nemoura* s.s.
4. Pregenital plate bilobed, reaching sternum IX (Fig 4A).       .*Sinonemura*
  - Pregenital plate rounded or quadrangular, not reaching sternum IX.       5
5. Cervical gills well developed on both sides of cervical sclerite.       *Zapada*
  - Cervical gills developed only on outer side of cervical sclerite, or both reduced to small nubs.        *Illiesonemoura*

**Comparative functional morphology of male epiproct.** Comparative analyses of the genitalia of several species belonging to *Nemoura* and the related genera, documented several paths of sperm transmission in the group.

*Sinonemura*: The sperm passes through the apical lobe of ventral sclerite that is stretched by the medial sclerites (Fig 3B, 3C and 3D).

*Zapada*: The sperm passes through the apical lobe of ventral sclerite without any supporting sclerites, but a central ductus can be seen in cleared specimens (Fig 5E and 5F).

*Illiesonemoura*: The two species groups clearly differ in type of sperm transfer: in case of the *pakistani* species group (Fig 6A and 6B), sperm transfer occurs similar to that of *Sinonemura*, whereas in the *polystigma* group, the sperm passes through the dorsal fold of the dorsal sclerite without any supporting sclerites (Fig 6C, 6D, 6E and 6F).

*Nemoura*: In the type species and those that have an apical sclerite (sensu stricto groups), the sperm passes through the apical lobe of ventral sclerite that is erected by the apical sclerites (Fig 5A, 5B, 5C and 5D). Among those species lacking an apical sclerite (sensu lato groups), sperm transfer is again different, though incompletely studied among its members. One special case is the *N*. *ovocercia* species group where sperm passes through a special modification called the cone-shaped projection [33].

In regard to the female inner genitalia, there is less variability and there appears no lock-and-key agreement with the male epiproct structure, but females of *Nemoura* s.s. have an inner sclerite that may connect with the apical sclerite of the epiproct during mating (Fig 4E and 4F).

### Zoogeography

**Fig 7:**
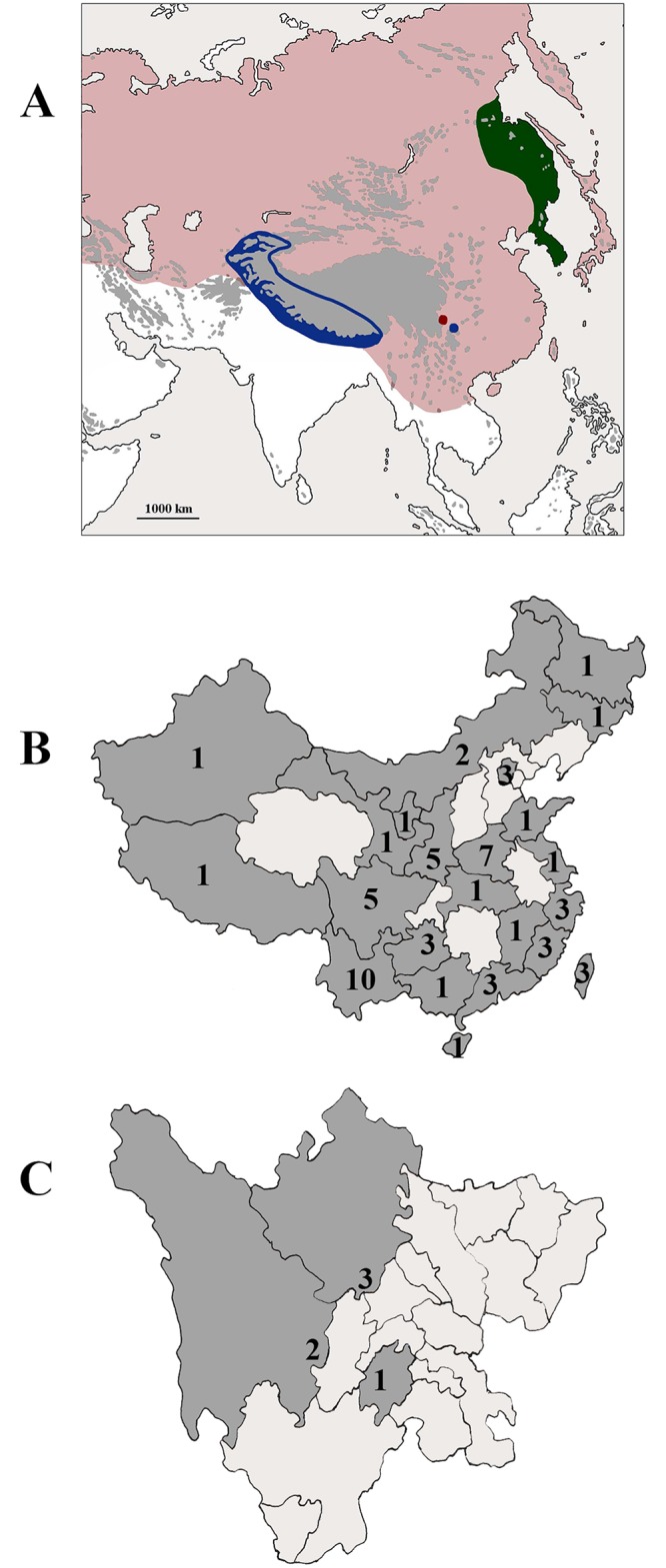
Asian distribution of genera *Illiesonemoura* Baumann, 1975, *Nemoura* Latreille, 1796, *Sinonemura* gen. n. and *Zapada* Ricker, 1952. (A) Distribution in Asia: blue: *Illesonemoura*; pink: *Nemoura*; red: *Sinonemura*; green: *Zapada*. Areas above 2000 meters shaded in grey. (B) Species number of *Nemoura* in provinces, regions and municipalities of China. Provinces and municipalities without records shaded in paler grey. (C) Known Nemourinae occurences in Sichuan Province: 1: *I*. *tuberosa*, *N*. *cochlocercia*, *N*. *furcocauda*, *N*. *janeti*; 2: *N*. *sichuanensis*, *N*. *stellata*; 3: *S*. *balangshana*. Cities and prefectures without records shaded in paler grey.

Among the four Asian genera belonging to the *Nemoura* genus group studied here, the Holarctic and Oriental genus *Nemoura* is widely distributed both in the Palaearctic and Oriental realm of the continent, but the *Nemoura* s.s. species are mostly distributed in the Palaearctic [2]. Alternatively, the Nearctic and East Palaearctic *Zapada* is known only from the Russian Far East and Korea in Asia [53], and *Sinonemura* is hitherto known only from Sichuan Province of southwest China, on the northern border of the Oriental realm. The genus *Illiesonemoura*, as currently understood comprised of the *polystigma* and *pakistani* species groups, is distributed in the Himalayan system, ranging from the Hindu Kush to the Qinghai-Tibet Plateau. An additional species from Taiwan was also assigned to the genus [9], but belongs to the *N*. *ovocercia* species group, and we reassign it to *Nemoura* herein: *Nemoura bispinosa* Kawai, 1968 [21] **comb. rev.** The Asian distribution of the four genera is displayed on Fig 7A.

Species assigned to the genus *Nemoura* (both in sensu stricto and sensu lato) can be found in most provinces of China (Fig 7B), while *Illiesonemoura* and *Sinonemoura* are currently known only from Sichuan Province (Fig 7C). This Province is located in southwestern of China occupying most of the Sichuan Basin and the easternmost part of the Tibetan Plateau between the Jinsha River on the west, the Daba Mountains in the north, and the Yungui Plateau to the south. Eastern Sichuan Province borders the Chinese city of Chongqing, and borders Yunnan and Guizhou provinces in the south. Tibet borders Sichuan Province to the west and to the north by Shaanxi, Gansu, and Qinghai provinces [61]. Three genera and seven species of subfamily Nemourinae are known to occur in Sichuan Province. The first study on the subfamily Nemourinae from Sichuan Province was by Wu, describing *I*. *tuberostyla* (Wu, 1962) [46] from Qingyin Pavillion of Mount Emei. *Nemoura cochleocercia* Wu, 1962 [46], *N*. *janeti* Wu, 1938 [43] and *N*. *furcocauda* Wu, 1973 [47] were reported from the same site [28,30,62]. Later, two new species *N*. *sichuanensis* Li & Yang, 2006 [25] and *N*. *stellata* Li & Yang, 2008 [27] were described from Luding County in Tibetan Autonomous Prefecture of Garzê. *Sinonemura balangshana* is the seventh Nemourinae species reported from Sichuan Province herein.

## Discussion

Recent molecular analyses and comparative functional morphology studies indicated that the Holarctic and Oriental genus *Nemoura* is paraphyletic, containing several different lineages [8]. We argued that the variability of the *Nemoura* terminalia is derived from different development of structures responsible for sperm transfer. To generate our generic concept about the *Nemoura* genus group, we used primarily genital characters that are directly involved in mating. Somatic characters that can be used for generic distinction in most Nemouridae, e.g. gill arrangement, seems to be of less use in this group.

Both morphological and molecular studies supported the distinction of the new genus *Sinonemura* that is herein described as a monotypic taxon. Possibly, the *pakistani* species group of the disputed genus *Illiesonemoura* may also belongs to *Sinonemura*, however, due no specimens of the *pakistani* group were available for molecular analyses, we left this question open. Though, *Illiesonemoura* is synonymous with *Nemoura* or not, it’s type species, *I*. *punctata* (Jewett, 1958) [63] belongs to the *polystigma* species group sensu Aubert [56], and this species can be well differentiated from the *pakistani* species group by lacking the parallel stick-like sclerites.

A sensu stricto diagnosis is applied for *Nemoura*, referring on the presence of apical sclerite in the male epiproct. Several Asian and a few Mediterranean species groups will require new generic placement, but herein we retrain these in *Nemoura* sensu lato. The Asian distribution of Nemourinae outlines is the center of genus level diversity in broad sense in the Himalayan region (Fig 7).

## Supporting information

S1 File Materials examined for comparative morphological analysis. (DOC)
