# Supplementary material for: A remarkable new genus and species of Nemourinae (Plecoptera, Nemouridae) from Sichuan, China, with systematic notes on the related genera
Source: PLoS One. 2020 Mar 4;15(3):e0229120. doi: 10.1371/journal.pone.0229120 (PMC7055821; doi:10.1371/journal.pone.0229120)
Supplement: S1 File — (DOC) [file pone.0229120.s001.doc]

**S1 File. Materials examined for comparative morphological analysis.**

*Nemoura* genus group:

*Illiesonemoura atripes* (Aubert, 1959): Pakistan, Gittidas, 11890 ft., 14.vii.1953, leg. F. Schmid: 1♀ paratype (ZML).

*Illiesonemoura battakundi* (Aubert, 1959): Pakistan, Lal Pani, 10-12000 ft., 2-3.ix.1953, leg. F. Schmid: 1♂ 1♀ paratypes (ZML).

*Illiesonemoura besali* (Aubert, 1959): Pakistan, Besal, 10715 ft., 24.vii.1953, leg. F. Schmid: 1♂ 1♀ paratypes (ZML).

*Illiesonemoura lilami* (Aubert, 1959): Pakistan, Kel, 6544 ft., 16.viii.1953, leg. F. Schmid: 1♂ paratype (ZML).

*Illiesonemoura pakistani* (Aubert, 1959): Pakistan, Battakundi, 9000 ft., 6.vii.1953, leg. F. Schmid: 1♂ 1♀ paratypes (ZML).

*Illiesonemoura polystigma* (Aubert, 1959): Pakistan, Besal, 10715 ft., 8-9.vii.1953, leg. F. Schmid: 1♂ 1♀ paratypes (ZML).

*Nemoura s.s. arctica* Esben-Petersen, 1910: Canada, Ontario, Grey county, Owen Sound, Inglis Falls, rheocrene spring, 04.vi.1985, leg. B.J. Sinclair: 2♂ 2♀, 1 larva (HNHM).

*Nemoura s.s. dubitans* Morton, 1894: Romania, Maramureș county, Igniș Mts, Desești-Stațiunea Izvoare, brook at the ecofarm N of Plesca, 850 m, N 47°49.507’ E 23°44.711’, 01.v.2009, leg. Z. Fehér, D. Murányi, A. Varga: 6♂ 6♀ (HNHM).

*Nemoura s.s. cambrica* Stephens, 1835: Slovakia, Banskobystrický region, Poľana Mts, Hrochoť, boggy forest brook at Beňova dolina, 550 m, N 48°39.069’ E 19°20.526’, 18.iv.2012, leg. T. Kovács, D. Murányi, T. Szederjesi: 5♂ (HNHM).

*Nemoura s.s. cercispinosa* Kawai, 1960: Japan, Ehime prefecture, Saijo city, Tuchigoya, forest seep and brook along Ishizuchi Skyline road, 1450 m, N 33°45.322’ E 133°09.436’, 22.v.2016, leg. H. Hosokawa, D. Murányi, S. Yamano: 2♂ 2♀ (HNHM).

*Nemoura s.s. cinerea cinerea* (Retzius, 1783): Hungary, Veszprém county, Bakonyalja, Pápa-Tapolcafő, Kalapács-ér Brook, 225 m, N 47°15.936’ E 17°32.803’, 27.iv.2014, leg. D. Murányi: 4♂ 2♀, 2 larvae (HNHM).

*Nemoura s.s. flexuosa* Aubert, 1949: Hungary, Pest county, Börzsöny Mts, Szokolya-Királyrét, Nagyvasfazék Stream, 255 m, N 47°53.672’ E 18°58.706’, 31.iii.2012, leg. D. Murányi: 2♂ 1♀ (HNHM).

*Nemoura s.s. fulva* (Šámal, 1921): Japan, Nagano prefecture, Shiojiri city, Narai, Narai River at the village, 940 m, N 35°58.194’ E 137°49.041’, 09.iv.2016, leg. D. Murányi, B. Páll-Gergely, S. Yamano: 4♂ 1♀ (HNHM).

*Nemoura s.s. fusca* Kis, 1963: Romania, Maramureș county, Igniș Mts, Săpănța, open springbrook above Sipot Waterfall, 650 m, N 47°55.865’ E 23°38.281’, 01.v.2009, leg. Z. Fehér, D. Murányi, A. Varga: 1♂ 1♀ (HNHM).

*Nemoura s.s. naraiensis* Kawai, 1954: Japan, Ehime prefecture, Saijo city, Tuchigoya, forest seep and brook along Ishizuchi Skyline road, 1450 m, N 33°45.322’ E 133°09.436’, 22.v.2016, leg. H. Hosokawa, D. Murányi, S. Yamano: 1♂ 2♀ (HNHM).

*Nemoura s.s. cercispinosa* Kawai, 1960: Japan, Ehime prefecture, Saijo city, Tuchigoya, forest seep and brook along Ishizuchi Skyline road, 1450 m, N 33°45.322’ E 133°09.436’, 22.v.2016, leg. H. Hosokawa, D. Murányi, S. Yamano: 2♂ 2♀ (HNHM).

*Nemoura s.s. papilla* Okamoto, 1922: China, Beijing, Wulingshan, Sixianjiaojie, 08.ix.2012, leg. W.C. Chang: 3♂ (HNHM).

*Nemoura s.s. sciurus* Aubert, 1949: Hungary, Borsod-Abaúj-Zemplén county, Rudabányai Mts, Szőlősardó, Rét Stream, 195 m, N 48°26.598’ E 20°39.683’, 11.iv.2014, leg. D. Murányi: 3♂ 2♀ (HNHM).

*Nemoura s.s. uenoi* Kawai, 1954: Japan, Kagawa prefecture, Takamatsu city, Sanuki Mts, Kaminishi, Koide Stream, 400 m, N 34°07.172’ E 134°04.380’, 02.iv.2016, leg. D. Murányi, S. Yamano: 2♂ 1♀ (HNHM).

*Nemoura s.s. uncinata* Despax, 1934: Albania, Kolonjë district, Grammos Mts, swampy springs and open brooks, 1940 m, N 40°19.907’ E 20°44.586’, 11.v.2014, leg. T. Kovács, D. Murányi: 2♂ 1♀ (HNHM).

*Nemoura s.l. akagii* Kawai, 1960: Japan, Kyoto prefecture, Kyoto city, Kita ward, Takagamine, Tenjin Stream and its tributary, 150 m, N 35°03.369’ E 135°43.440’, 05.iv.2016, leg. D. Murányi, S. Yamano: 1♂ 1♀ (HNHM).

*Nemoura s.l. chinonis* (Okamoto, 1922): Japan, Kagawa prefecture, Takamatsu city, Sanuki Mts, Kaminishi, Koide Stream, 400 m, N 34°07.172’ E 134°04.380’, 02.iv.2016, leg. D. Murányi, S. Yamano: 2♂ 1♀ (HNHM).

*Nemoura s.l. geei* Wu, 1929: South Korea, Gangwon province, Yangyang-gun, Seorak Mts, Hangae-ryeong Pass, brook in deciduous forest, 875 m, N 38°05.481’ E 128°24.267’, 09.ix.2010, leg. L. Forró, Gy. Makranczy, D. Murányi, S.J. Park, J.D. Yoon: 3♂ 7♀ (HNHM).

*Nemoura s.l. lacustris* Pictet, 1865: Spain, Castille and León, Zamora province, Sanabria Lake, 1020 m, N 42°07’ W 6°43’, 05.v.1991, leg. G. Vinçon: 2♂ 1♀ (HNHM).

*Nemoura s.l. longicercia* Okamoto, 1922: Japan, Ehime prefecture, Kumakogen town, Tuchigoya, forest brook along Ishizuchi Skyline road, 1500 m, N 33°45.204’ E 133°08.857’, 15.x.2015, leg. Y. Kaetani, D. Murányi, S. Yamano: 3♂ 5♀, 8 larvae (HNHM).

*Nemoura s.l. ovocercia* Kawai, 1960: Japan, Ehime prefecture, Toōn city, Kamihayashi, cascading rocky brook along road No.209, 820 m, N 33°43.691’ E 132°53.511’, 03.v.2017, leg. D. Murányi: 2♂ 5♀ (HNHM).

*Nemoura s.l. rotundprojecta* Du & Zhou, 2008: China, Shaanxi, Hangzhong city, Foping county, Qinling Mts, brook in Lover’s valley, by Foping old town, 885 m, N 33°31.838’ E 107°59.432’, 21.iv.2018, leg. W.H. Li, R.R. Mo, D. Murányi: 2♂ 1♀, 2 larvae (HNHM).

*Nemoura s.l. stratum* Kawai, 1966: Japan, Nagano prefecture, Shiojiri city, Narai, forest brook at Shinmeigu Shrine, 975 m, N 35°57.937’ E 137°48.583’, 09.iv.2016, leg. D. Murányi, B. Páll-Gergely, S. Yamano: 1♂ (HNHM).

*Zapada cinctipes* (Banks, 1897): USA, California, San Diego county, Palomar Mts, Iron Springs Creek along road S-6, 1490 m, N 33°20.004’ W 116°52.357’, 11.i.2012, leg. B.C. Kondratieff, D. Murányi, C.R. Nelson, J.B. Sandberg: 2♂ 3♀ (HNHM).

Other Nemourinae genera:

*Lednia tumana* (Ricker, 1952): USA, Montana, Glacier county, Glacier N.P., Logan Pass, base of Mt. Clements, behind moraine on snow, 16.x.1997, leg. J. Giersch: 1♂ 1♀ (HNHM).

*Nanonemoura wahkeena* (Jewett, 1954): USA, Oregon, Multnomah county, Wahkeena Creek and seeps, 17.iv.1984, leg. G.R. Fiala: 1♂ 1♀ (HNHM).

*Nemurella pictetii* (Klapálek, 1900): Hungary, Heves county, Mátra Mts, Mátraszentimre-Galyatető, Galya-csurgó Spring, 895 m, N 47°54.882’ E 19°55.542’, 21.v.2015, leg. T. Kovács, D. Murányi, G. Puskás: 10♂ 3♀ (HNHM).

*Ostrocerca foersteri* (Ricker, 1943): USA, Oregon, Clackamus county, Salmon River above Welches, 25.v.1990, leg. G.R. Fiala: 2♂ 2♀ (HNHM).

*Paranemoura perfecta* (Walker, 1852): USA, West Virginia, Pocahontas county, headwaters of Sugar Creek, 24.v.1994, leg. R.F. Kirchner, B.C. Kondratieff: 2♂ 2♀, 2 larvae (HNHM).

*Podmosta decepta* (Frison, 1942): Canada, Alberta, Waterton Lakes N.P., Cameron Creek, 21.vii.1966, leg. A.R. Gaufin: 2♂ 2♀ (HNHM).

*Prostoia completa* (Walker, 1852): USA, Missouri, Webster county, Hwy 14 near Evans, Bryant Creek, 22.ii.1972, leg. R.W. Baumann, S.W. Szczytko: 2♂ 2♀, 1 larva (HNHM).

*Shipsa rotunda* (Claassen, 1923): Canada, Labrador, Walsh River, inlet of Long Lake, 11.vi.1997, leg. R.W. Baumann, B.C. Kondratieff, C.R. Nelson: 2♂ 2♀, 2 larvae (HNHM).

*Soyedina vallicularia* (Wu, 1923): USA, Indiana, Parke county, Turkey Run State Park, spring seep, 23.iii.1975, leg. R.W. Baumann, O.S. Flint: 2♂ 2♀, 1 larva (HNHM).

*Visoka cataractae* (Neave, 1933): USA, Montana, Missoula county, Grant Creek near Missoula, 15.iv.1972, leg. R.A. Haick: 2♂ 2♀ (HNHM).

Amphinemurinae genera:

*Amphinemura banksi* Baumann & Gaufin, 1972: USA, Utah, Utah county, Wasatch Mts, South Fork of American Fork River at Altamount Campground, 2190 m, N 40°26.145’ W 111°38.147’, 14.ix.2011, leg. R.W. Baumann, S.M. Clark, D. Murányi: 1♂ (HNHM).

*Amphinemura flavostigma* Okamoto, 1922: Japan, Ehime prefecture, Kumakogen town, Nishidani, upper section of Myogatani River along road No.328, 900 m, N 33°32.922’ E 132°56.904’, 20.v.2016, leg. H. Hosokawa, D. Murányi, S. Yamano: 2♂ (HNHM).

*Amphinemura hamiornata* Li & Yang, 2008: China, Guangxi, Shangsi county, Shiwandashan National Forest Park, Pearl River above tourist route bridge, 375 m, N 21°53.913’ E 107°54.283’, 27.iii.2015, leg. J. Kontschán, J.N. Li, S. Li, W.H. Li, D. Murányi, G.Q. Wang: 5♂ 4♀ (HNHM).

*Amphinemura megaloba* (Kawai, 1960): Japan, Kochi prefecture, Niyodogawa town, Kawado, Choja River along road No.439, 190 m, N 33°31.573’ E 133°08.083’, 17.iii.2017, leg. D. Murányi: 5♂ 2♀ (HNHM).

*Amphinemura sulcicollis* (Stephens, 1836): Montenegro, Mojkovac municipality, Sinjajevina Mts, Gornja Polja, Zoljski Ljevak Stream above the village, 880 m, N 42°57.808’ E 19°31.597’, 14.vi.2012, leg. Z. Fehér, T. Kovács, D. Murányi: 2♂ 1♀ (HNHM).

*Indonemoura scalprata* (Li & Yang, 2007): China, Guangxi, Wuming county, Liangjiang town, Neichao, Neichao River above Neichao Ming Hotel, 220 m, N 23°29.664’ E 108°21.622’, 24.iii.2015, leg. J.N. Li, S. Li, W.H. Li, D. Murányi: 1♂ 3♀ (HNHM).

*Malenka californica* (Claassen, 1923): USA, California, Los Angeles county, San Gabriel Mts, Soldier Creek at Falling Springs, 1205 m, N 34°18.081’ W 117°50.294’, 13.i.2012, leg. B.C. Kondratieff, D. Murányi, C.R. Nelson: 5♂ 6♀ (HNHM).

*Protonemura brevistyla* (Ris, 1902): Romania, Brașov county, Făgăraș Mts, Dejani, upper section of Dejani Stream, 1580 m, N 45°35.715’ E 24°56.374’, 30.viii.2012, leg. T. Kovács, D. Murányi, J. Oláh: 2♂ 6♀ (HNHM).

*Sphaeronemoura separata* Li, Murányi & Yang, 2014: China, Henan, Luanchuan county, Chongdugou Scenic Area, upper tributary of Jinji River, 1200 m, N 33°94.08’ E 111°72.78’, 20.viii.2013, leg. W.H. Li: 1♂ 1♀, 1 larva paratypes (HNHM).
